# Pretreatment Prediction of Tumor Recurrence in Breast Cancer After Neoadjuvant Systemic Therapy Using Machine Learning With Clinical and CT Radiomics Features

**DOI:** 10.1002/kjm2.70288

**Published:** 2026-08-26

**Authors:** Huei‐Yi Tsai, Jo‐Ching Wang, Wei‐Shiuan Chung, Jui‐Sheng Hsu, Shu‐Yen Lin, Ming‐Feng Hou, Ming‐Chung Chou

**Affiliations:** ^1^ Graduate Institute of Clinical Medicine, College of Medicine Kaohsiung Medical University Kaohsiung Taiwan; ^2^ Department of Medical Imaging, Kaohsiung Medical University Hospital Kaohsiung Medical University Kaohsiung Taiwan; ^3^ Center for Big Data Research Kaohsiung Medical University Kaohsiung Taiwan; ^4^ Department of Medical Research Kaohsiung Medical University Hospital Kaohsiung Taiwan; ^5^ Department of Medical Imaging Kaohsiung Municipal Siaogang Hospital Kaohsiung Taiwan; ^6^ Biomedical Artificial Intelligence Academy Kaohsiung Medical University Kaohsiung Taiwan; ^7^ Department of Biomedical Science and Environmental Biology, College of Life Science Kaohsiung Medical University Kaohsiung Taiwan; ^8^ Department of Medical Imaging and Radiological Sciences Kaohsiung Medical University Kaohsiung Taiwan

**Keywords:** breast cancer, computed tomography, machine learning, radiomics, recurrence

## Abstract

This study aimed to develop machine learning models for predicting tumor recurrence in breast cancer before neoadjuvant systemic therapy (NST) by integrating clinical and radiomic features derived from pretreatment computed tomography (CT). We retrospectively enrolled 235 patients with 237 breast tumors who underwent contrast‐enhanced CT before NST. Datasets were randomly divided into five‐fold training and testing sets using semi‐random partitioning to ensure similar clinical characteristics between the two subsets. Subsequently, a nested five‐fold cross‐validation was performed to develop a recurrence prediction model using three machine learning algorithms across clinical, radiomics, and integrated models. The performance of prediction models was compared using the area under the receiver‐operating characteristic curve (AUC), and the best clinical and radiomics models were further integrated to develop the final model. Kaplan–Meier analysis with a log‐rank test was conducted to compare survival curves between high‐ and low‐risk groups stratified by the prediction models. The comparisons demonstrated that the random survival forest (RSF) clinical model (mean AUC = 0.755) and the Cox‐least absolute shrinkage and selection operator (Cox‐LASSO) radiomics model (mean AUC = 0.636) outperformed other machine learning algorithms. The integration of the clinical (RSF) and radiomics (Cox‐LASSO) models achieved a mean AUC of 0.777 in predicting tumor recurrence. The log‐rank analysis revealed significant differences in the survival curves between the high‐ and low‐risk groups stratified by the integration model on the testing sets. In conclusion, the integration of clinical and CT‐based radiomics models was helpful for the pretreatment prediction of tumor recurrence in patients with breast cancer after NST.

## 1 Introduction

Breast cancer is the most common malignancy among women and the second most common cancer globally, with an estimated 2.3 million new cases and 670,000 deaths reported in 2022 [1]. Advances in screening and treatment have raised the overall 5‐year survival rate to approximately 90%, particularly for localized disease [2]. However, a substantial proportion of patients experience disease recurrence, either as local relapse, distant metastasis, or both, resulting in significantly worse outcomes. Median survival after recurrence ranges from approximately 18–49 months, with 5‐year overall survival rates varying between 19.9% and 77%, based on the burden and severity of adverse prognostic factors [3, 4, 5].

The identification of risk factors for cancer recurrence is essential for guiding individualized treatment and follow‐up strategies in patients with primary breast cancer. Previous studies have demonstrated that recurrence is associated with larger tumor size, axillary lymph node metastasis [6], advanced stage at diagnosis, and aggressive molecular subtypes, including human epidermal growth factor receptor 2 (HER2)‐positive and triple‐negative breast cancer [7, 8]. Further, younger age (< 40 years) and positive surgical margins have been reported as features of recurrence [9]. The absence of a pathological complete response is a strong predictor of recurrence among patients receiving neoadjuvant systemic therapy (NST) [10, 11, 12, 13]. Furthermore, Zdanowski et al. showed that among patients receiving NST, those who have extremely dense breast tissue on mammography are at increased risk of recurrence and breast cancer‐specific mortality [14].

To improve the prediction of tumor recurrence, several studies have applied machine learning and deep learning approaches using clinical, pathological, and immunohistochemical data of breast tumors, with reported models achieving area under the receiver‐operating characteristic (ROC) curve (AUC) ranging from 0.69 to 0.92 [15, 16, 17, 18]. Other investigations employed pretreatment imaging modalities to predict recurrence, including ultrasound [19, 20], mammography [20], magnetic resonance imaging (MRI) [21, 22, 23], and positron emission tomography (PET)/computed tomography (CT) [24], with AUCs ranging from 0.67 to 0.99. However, to date, no study has used pretreatment contrast‐enhanced CT (CECT) images and combined clinical data to collectively develop a predictive model for tumor recurrence in patients with breast cancer after NST.

In clinical practice, CECT is routinely performed to assess potential tumor metastasis in patients with breast cancer before NST [25]. However, whether the integration of clinical variables and imaging features extracted from CECT can effectively develop a machine learning model for predicting tumor recurrence after NST remains unclear. We hypothesized that combining clinical data with CECT‐based radiomic features could help develop an accurate predictive model for recurrence. Accordingly, this study aimed to construct machine learning models for predicting tumor recurrence in patients with breast cancer after NST using both clinical and CECT‐derived features.

## 2 Materials and Methods

The local Institutional Review Board of Kaohsiung Medical University Hospital approved this retrospective and single‐centered study (protocol code: KMUHIRB‐E(I)‐20200406). Informed consent was waived due to the retrospective nature of the study.

### 2.1 Study Subjects

**FIGURE 1:**
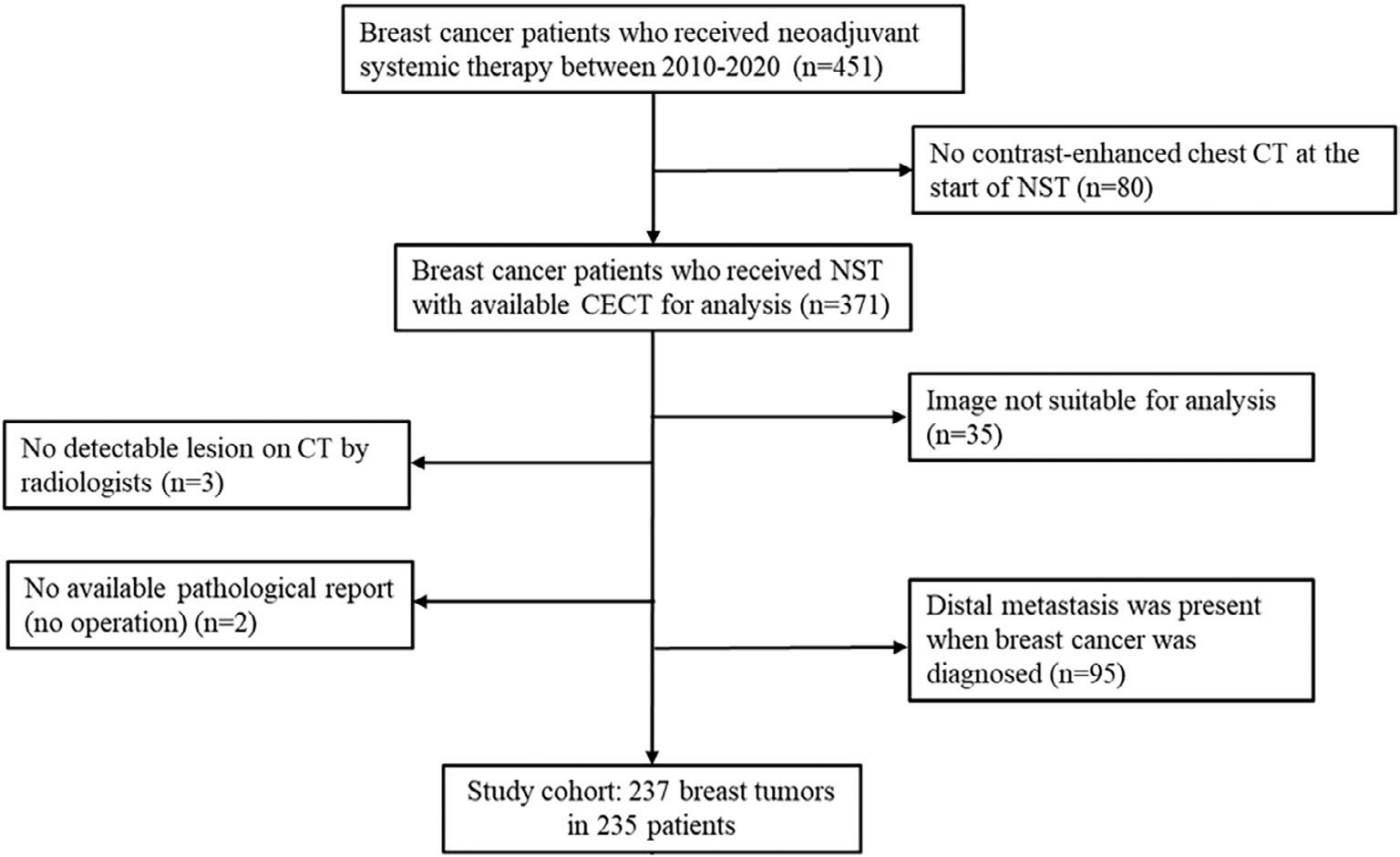
Flowchart of data collection illustrating that 235 patients with 237 breast tumors were successfully enrolled in this study. The patients were categorized into recurrence (*n* = 56, 23.5%) and non‐recurrence groups (*n* = 181, 76.4%) based on their diagnosis during the study period. CECT, contrast‐enhanced computed tomography; CT, computed tomography; NST, neoadjuvant systemic therapy.

We retrospectively enrolled 451 consecutive patients with breast cancer who underwent NST from January 2010 to December 2020. The treatment regimens were administered following the National Comprehensive Cancer Network guidelines [25]. The present study included patients with breast cancer who underwent a CECT scan either 2 months before NST or 1 day after NST and recorded their recurrence status during the study period. Exclusion criteria were (a) images unsuitable for analysis, (b) tumors undetectable by radiologists, (c) unavailable pathological reports, or (d) distant metastasis along with breast cancer. Finally, the study cohort successfully included 237 breast tumors in 235 patients (Figure 1).

### 2.2 Clinical Data

Clinical data, including age, clinical stage, cancer cell types (e.g., ductal, lobular, connective tissue), estrogen receptor (ER), progesterone receptor (PR), and HER2 status, were obtained to predict tumor recurrence after NST. Further, breast cancers were categorized into different subtypes based on hormone receptor (HR) and HER2 expression, including HR‐positive/HER2‐positive, HR‐positive/HER2‐negative, HER2‐positive, and triple‐negative. Table 1 summarizes the demographic characteristics of the enrolled patients with breast cancer.

**TABLE 1:** Comparison of clinical features between patients with or without recurrence (*N*, %).

	No recurrence	Recurrence	Total	
	*n* = 181 (76.4%)	*n* = 56 (23.6%)	*n* = 237 (100%)	p
Age (mean ± SD)	51.4 ± 9.7	50.5 ± 10.1	51.2 ± 9.8	0.567
Breast surgery				0.054
Total mastectomy	98 (54.1)	40 (71.4)	138	
Skin or nipple‐sparing mastectomy	22 (12.2)	6 (10.7)	28	
Breast conserving surgery (BCS)	61 (33.7)	10 (17.9)	71	
Breast surgery (binary classification)				0.024
Total mastectomy	120 (66.3)	46 (82.1)	166	
Breast conserving surgery (BCS)	61 (33.7)	10 (17.9)	71	
Axillary LN surgery				0.006
ALND (dissection)	102 (56.4)	43 (76.8)	145	
SLNB (biopsy)	79 (43.6)	13 (23.2)	92	
Flap reconstruction				0.238
Yes	165 (91.2)	48 (85.7)	203	
No	16 (8.8)	8 (14.3)	24	
Clinical staging				0.002
1	16 (8.8)	0	16	
2	104 (57.5)	24 (42.9)	128	
3	61 (33.7)	32 (57.1)	93	
pCR				0.01
No	140 (77.3)	52 (92.9)	192	
Yes	41 (22.7)	4 (7.1)	45	
Tumor subtype				0.003
HR(+)_HER2(+)	45 (24.9)	12 (21.4)	57	
HR(+)_HER2(−)	85 (47.0)	18 (32.1)	103	
HER2‐enriched	30 (16.6)	6 (10.7)	36	
Triple‐negative	19 (10.5)	17 (30.4)	36	
Histology				0.926
IDC	171 (94.5)	53 (94.6)	224	
Others	10 (5.5)	3 (5.4)	13	

Abbreviations: HER2, human epidermal growth factor 2; IDC, invasive carcinoma of no special type; pCR, pathological complete response; SD, standard deviation.

### 2.3 Imaging Data

The CECT images of patients with breast cancer were acquired from different CT scanners, including Siemens (SOMATOM Definition, SOMATOM Definition AS, SOMATOM Definition Flash, and Sensation 64), GE (Optima CT 660, Discovery CT 750 HD, BrightSpeed S, and LightSpeed 16), Philips (Brilliance 64), and Toshiba (Aquilion, Aquilion ONE, and Aquilion PRIME). Each patient underwent a CECT scan after intravenous iodine contrast injection following the routine standard protocol of our hospital. The contrast delay time was approximately 80–90 s after injection, and the scan covered the entire chest region, and images were reconstructed with a 512 × 512 matrix size, 5 mm slice thickness, and no gap. No additional interpolation and intensity normalization were performed for all CT images.

### 2.4 Image Segmentation and Feature Extraction

**FIGURE 2:**
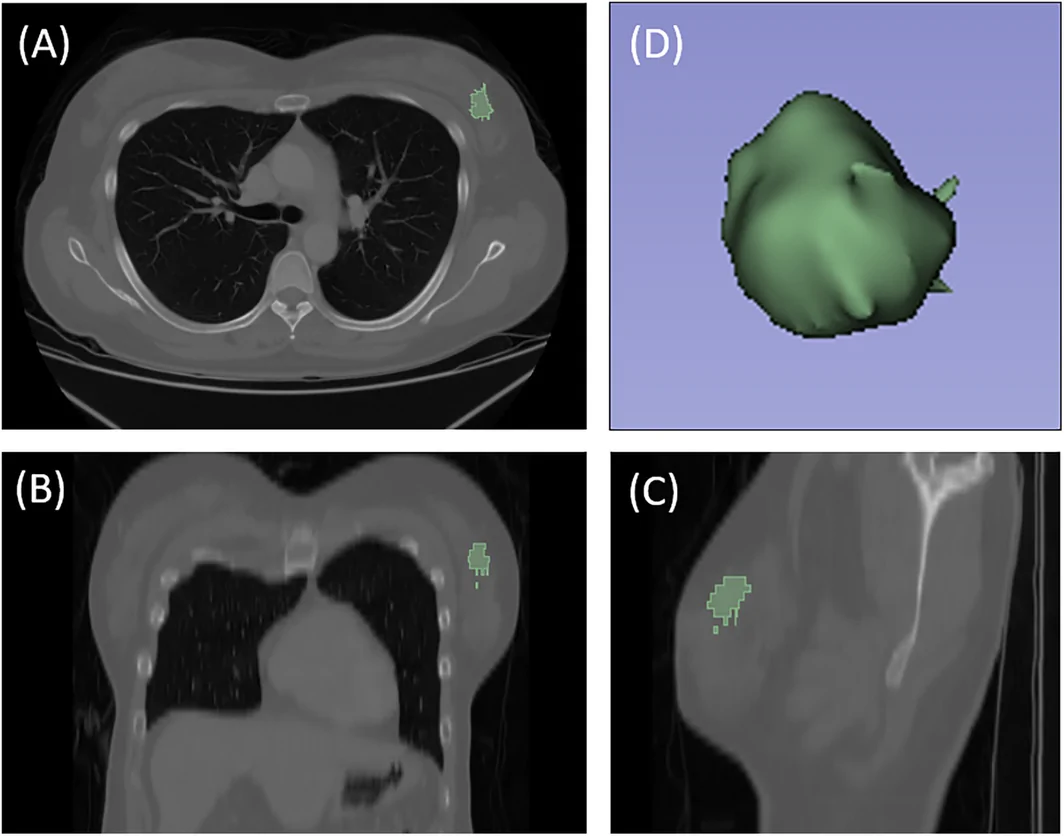
Semi‐automatic image segmentation using the GrowCut method in axial (A), coronal (B), and sagittal (C) images. After image segmentation, the three‐dimensional contour of the breast tumor was obtained for radiomics analysis (D).

Breast tumor segmentation was performed based on axial images using the GrowCut semi‐automatic segmentation method of 3D‐Slicer software (Slicer 4.11.20210226) [26], following the same protocol as our previous study on the same cohort (Figure 2) [27]. A third‐year resident (T.Y.T.) in the radiology department first processed the images, and two radiologists with consensus agreement (H.Y.T. and C.H.W., with 17 and 4 years of experience, respectively) confirmed the contour of each case. Radiomic features were automatically extracted using PyRadiomics, an open‐source Python package, version 3.0.1 [28]. This study automatically extracted 107 radiomics features of seven classes, comprising 14 shape features, 18 first‐order features, and 75 textural features of the gray‐level dependence matrix, gray‐level run length matrix, gray‐level size zone matrix, and neighboring gray tone difference matrix (Table S1).

### 2.5 Inter‐Rater Reliability

In the present study, two radiologists (H.Y.T. and C.H.W.) separately performed tumor masking for 20 randomly selected study participants to assess inter‐reader reliability. The Dice coefficient was used to calculate inter‐rater reliability, and cases with Dice scores lower than 0.7 were resolved by consensus with a third senior radiologist (J.S.H., with 25 years of experience). The semi‐automatic segmentation tool yielded good concordance of lesion segmentation, with an average Dice score of 0.86 ± 0.08. The reproducibility of radiomic features was further investigated by calculating the intraclass correlation coefficient (ICC) of the two volumes of interest masked by the two radiologists for the same lesion. Moreover, this procedure facilitated the alignment of consensus between the two readers before full‐cohort segmentation.

Afterward, a third‐year radiology resident (T.Y.T.) performed segmentation on the full cohort of 237 breast tumors, and two radiologists (H.Y.T. and C.H.W.) confirmed the resulting contours through consensus review of every case. Finally, all 237 final masks were used for radiomic feature extraction. Moreover, CT images were acquired from different scanners; thus, the radiomics features were further harmonized using ComBat harmonization to remove possible variations (batch effects) from multi‐scanner datasets [29].

### 2.6 Machine Learning

The dataset was randomly divided into five folds at the patient level employing a semi‐random partitioning method [30], which ensured that the training (number of recurrence = 48) and testing (number of recurrence = 8) subsets of each fold maintained similar demographic characteristics without significant differences, including age, clinical stage, cancer cell types, ER, PR, HER2 status, and tumor recurrence. The tumor recurrence rate was 23.6% in the study cohort, which could introduce bias in model training; thus, we applied the synthetic minority over‐sampling technique (SMOTE) to balance the tumor recurrence in the training folds of the training set [31]. For the clinical model, the input features included age, clinical stage, cancer cell type, ER, PR, and HER2 obtained before treatment. For the CT radiomics model, the input features included 107 radiomics features extracted from breast tumors and were selected based on ICC thresholds of 0.70, 0.75, 0.80, 0.85, 0.90, and 0.95 for comparison purposes. For the integration model, the best clinical and radiomics models were stacked to collectively predict the recurrence by averaging the outputs of the two best models. Subsequently, three machine learning algorithms were employed to train the prediction model for tumor recurrence, including random survival forest (RSF), Cox‐proportional hazards (Cox‐PH), and Cox‐least absolute shrinkage and selection operator (Cox‐LASSO) models.

To evaluate how clinical, radiomics, and integrated data impact the accuracy of predicting tumor recurrence, machine learning algorithms were trained using clinical, radiomics, and integrated data, respectively. For model training, hyperparameter tuning was performed using a nested five‐fold cross‐validation, and model performance was assessed using the five testing sets. After developing the final model, feature importance was assessed by calculating the SHapley Additive exPlanations (SHAP) value of each variable of the integration model on the testing sets, and features were ranked based on their percentage change in accuracy. Further, Kaplan–Meier analysis with a log‐rank test was conducted to compare the recurrence‐free survival curves between the high‐ and low‐risk groups. The machine learning prediction models were trained using the scikit‐learn library of Python software version 3.10.

### 2.7 Statistical Analysis

Differences in demographic characteristics between the training and testing sets were investigated using the Pearson *χ* ^2^ test and the two‐sample *t* test. ROC analysis was conducted to compare the sensitivity, specificity, and AUC among the prediction models. The results were expressed as mean ± standard deviation with 95% confidence interval (CI). Kaplan–Meier analysis with a log‐rank test was used to compare recurrence‐free survival curves. The comparison was considered significant if the *p*‐value was less than 0.05 with Bonferroni correction applied for multiple comparisons.

## 3 Results

### 3.1 Demographic Characteristics of Enrolled Patients

Pathological analysis revealed that out of 237 tumors, 56 (23.6%) experienced tumor recurrence after surgical resection during the follow‐up period. The immunohistochemical analysis indicated that of 237 tumors, 107 (45%) and 60 (25%) were HR‐positive with and without HER2 positivity, respectively; 36 (15%) demonstrated HER2 overexpression, and 36 (15%) were classified as triple‐negative. The comparison revealed that patients with recurrence showed significantly higher rates of HR(+) and HER2(+), HR(+) and HER2(−), and HER2‐enriched tumors compared to those without recurrence, leading to a significant difference in tumor subtype between the two groups (Table 1).

In patients with tumor recurrence, the results indicated that the mean time to recurrence was 2.8 ± 2.4 years after surgical resection of tumors. Recurrent sites were primarily local (*n* = 10), distant (*n* = 9), and involved skeletal metastases (*n* = 8) (Table 2).

**TABLE 2:** Time to recurrence and tumor recurrence patterns (*N*, %).

Time to recurrence (year)	
Mean ± SD	2.8 ± 2.4
Minimum–maximum	0.67–11.82
Recurrence pattern	
Local recurrence	13 (23.2)
Regional LNs	6 (10.7)
Distant metastasis	30 (53.6)
Pulmonary	8 (14.3)
Hepatic	2 (3.6)
Skeletal	8 (14.3)
CNS	2 (3.6)
Multiple sites	10 (17.9)
Nonregional LNs	4 (7.1)
Local/regional+distant mets	3 (5.4)
Total	56 (100)

Abbreviations: CNS, central nervous system; LN, lymph node; mets, metastasis; SD, standard deviation.

### 3.2 Model Performance of Recurrence Prediction

In the clinical model, the comparison revealed that the RSF algorithm outperformed the other two machine learning algorithms in predicting tumor recurrence, achieving AUCs of 0.820 ± 0.056, 0.731 ± 0.015, and 0.732 ± 0.024 in the testing set at 1, 2, and 3 years post‐treatment, respectively (Table 3).

**TABLE 3:** Prediction performance of the clinical model between the three ML algorithms at 1, 2, and 3 years post‐treatment.

Clinical model	Training set			Testing set		
	AUC	Sensitivity	Specificity	AUC	Sensitivity	Specificity
Cox‐PH (1 year)	0.813 ± 0.026 (0.782–0.845)	0.891 ± 0.014 (0.874–0.909)	0.601 ± 0.034 (0.559–0.644)	0.772 ± 0.116 (0.588–0.956)	0.906 ± 0.188 (0.608–1.000)	0.556 ± 0.033 (0.503–0.608)
Cox‐PH (2 years)	0.782 ± 0.017 (0.761–0.803)	0.794 ± 0.039 (0.745–0.843)	0.637 ± 0.034 (0.595–0.679)	0.696 ± 0.062 (0.615–0.769)	0.732 ± 0.088 (0.623–0.842)	0.529 ± 0.018 (0.506–0.551)
Cox‐PH (3 years)	0.809 ± 0.018 (0.787–0.832)	0.777 ± 0.023 (0.749–0.806)	0.707 ± 0.048 (0.648–0.767)	0.707 ± 0.057 (0.632–0.773)	0.741 ± 0.107 (0.608–0.874)	0.600 ± 0.039 (0.552–0.648)
Cox‐LASSO (1 year)	0.810 ± 0.028 (0.775–0.845)	0.879 ± 0.028 (0.844–0.913)	0.598 ± 0.022 (0.571–0.625)	0.771 ± 0.113 (0.591–0.951)	0.865 ± 0.178 (0.581–1.148)	0.549 ± 0.023 (0.513–0.586)
Cox‐LASSO (2 years)	0.781 ± 0.017 (0.760–0.801)	0.798 ± 0.044 (0.743–0.853)	0.641 ± 0.033 (0.600–0.683)	0.693 ± 0.065 (0.611–0.774)	0.707 ± 0.099 (0.584–0.830)	0.523 ± 0.008 (0.513–0.532)
Cox‐LASSO (3 years)	0.809 ± 0.019 (0.785–0.833)	0.777 ± 0.028 (0.742–0.812)	0.707 ± 0.045 (0.651–0.763)	0.709 ± 0.054 (0.643–0.776)	0.716 ± 0.119 (0.569–0.863)	0.593 ± 0.031 (0.554–0.632)
RSF (1 year)	0.881 ± 0.023 (0.852–0.910)	0.965 ± 0.035 (0.922–1.000)	0.623 ± 0.039 (0.574–0.672)	0.820 ± 0.056 (0.730–0.910)	0.969 ± 0.062 (0.869–1.000)	0.549 ± 0.023 (0.513–0.586)
RSF (2 years)	0.838 ± 0.014 (0.820–0.855)	0.853 ± 0.025 (0.822–0.884)	0.664 ± 0.015 (0.645–0.683)	0.731 ± 0.015 (0.712–0.750)	0.837 ± 0.051 (0.774–0.900)	0.540 ± 0.025 (0.509–0.571)
RSF (3 years)	0.855 ± 0.009 (0.844–0.866)	0.830 ± 0.020 (0.805–0.856)	0.746 ± 0.022 (0.718–0.774)	0.732 ± 0.024 (0.703–0.761)	0.813 ± 0.063 (0.735–0.891)	0.580 ± 0.054 (0.513–0.646)

*Note:* The numbers in parentheses are 95% confidence intervals.

Abbreviations: AUC, area under the ROC curve; Cox‐LASSO, Cox‐least absolute shrinkage and selection operator; Cox‐PH, Cox‐proportional hazards; RSF, random survival forest.

In the radiomics model, the comparison indicated that the Cox‐LASSO algorithm outperformed the other two machine learning algorithms and achieved AUCs of 0.635 ± 0.168, 0.633 ± 0.138, and 0.666 ± 0.092 at an ICC threshold of ≥ 0.95 (23 radiomics features) in the testing set at 1, 2, and 3 years post‐treatment (Table 4).

**TABLE 4:** Prediction performance of the radiomics model between three machine learning algorithms with an ICC threshold of ≥ 0.95 at 1, 2, and 3 years post‐treatment.

Radiomics model	Training set			Testing set		
	AUC	Sensitivity	Specificity	AUC	Sensitivity	Specificity
Cox‐PH (1 year)	0.686 ± 0.019 (0.663–0.709)	0.674 ± 0.054 (0.606–0.741)	0.537 ± 0.013 (0.521–0.553)	0.609 ± 0.141 (0.384–0.833)	0.631 ± 0.125 (0.433–0.830)	0.519 ± 0.007 (0.507–0.530)
Cox‐PH (2 years)	0.745 ± 0.031 (0.706–0.783)	0.721 ± 0.046 (0.664–0.778)	0.594 ± 0.024 (0.564–0.623)	0.611 ± 0.113 (0.471–0.752)	0.671 ± 0.144 (0.493–0.850)	0.512 ± 0.039 (0.464–0.560)
Cox‐PH (3 years)	0.752 ± 0.040 (0.702–0.802)	0.730 ± 0.041 (0.679–0.781)	0.596 ± 0.039 (0.547–0.644)	0.644 ± 0.102 (0.517–0.771)	0.711 ± 0.142 (0.535–0.887)	0.510 ± 0.060 (0.435–0.585)
Cox‐LASSO (1 year)	0.698 ± 0.029 (0.662–0.734)	0.695 ± 0.063 (0.618–0.773)	0.541 ± 0.023 (0.512–0.570)	0.635 ± 0.168 (0.367–0.904)	0.704 ± 0.247 (0.311–1.000)	0.525 ± 0.027 (0.482–0.567)
Cox‐LASSO (2 years)	0.776 ± 0.022 (0.749–0.803)	0.755 ± 0.031 (0.717–0.794)	0.611 ± 0.016 (0.591–0.631)	0.633 ± 0.138 (0.462–0.804)	0.668 ± 0.208 (0.410–0.926)	0.524 ± 0.057 (0.453–0.595)
Cox‐LASSO (3 years)	0.772 ± 0.017 (0.751–0.793)	0.754 ± 0.029 (0.719–0.790)	0.614 ± 0.044 (0.559–0.669)	0.666 ± 0.092 (0.552–0.780)	0.722 ± 0.148 (0.539–0.905)	0.526 ± 0.058 (0.454–0.598)
RSF (1 year)	0.908 ± 0.042 (0.856–0.960)	0.996 ± 0.008 (0.986–1.000)	0.627 ± 0.034 (0.584–0.669)	0.616 ± 0.031 (0.566–0.665)	0.696 ± 0.200 (0.377–1.000)	0.525 ± 0.042 (0.459–0.591)
RSF (2 years)	0.941 ± 0.026 (0.909–0.974)	0.957 ± 0.012 (0.943–0.972)	0.726 ± 0.036 (0.681–0.771)	0.646 ± 0.054 (0.579–0.714)	0.719 ± 0.083 (0.615–0.823)	0.540 ± 0.018 (0.517–0.562)
RSF (3 years)	0.925 ± 0.029 (0.889–0.962)	0.950 ± 0.011 (0.936–0.963)	0.737 ± 0.046 (0.680–0.795)	0.603 ± 0.112 (0.464–0.742)	0.658 ± 0.142 (0.482–0.834)	0.443 ± 0.097 (0.323–0.563)

*Note:* The numbers in parentheses are 95% confidence intervals.

Abbreviations: AUC, area under the ROC curve; Cox‐LASSO, Cox‐least absolute shrinkage and selection operator; Cox‐PH, Cox‐proportional hazards; RSF, random survival forest.

In the integration model, the present study integrated the best clinical (RSF) and radiomics (Cox‐LASSO) models for predicting tumor recurrence. The comparison revealed that the integration model achieved AUCs of 0.793 ± 0.069, 0.762 ± 0.067, and 0.781 ± 0.082 at an ICC threshold of ≥ 0.95 in the testing sets at 1, 2, and 3 years post‐treatment, respectively (Table 5).

**TABLE 5:** Prediction performance of the integration model with an ICC threshold of ≥ 0.95 at 1, 2, and 3 years post‐treatment.

Integration model	Training set			Testing set		
	AUC	Sensitivity	Specificity	AUC	Sensitivity	Specificity
1 year	0.880 ± 0.032 (0.840–0.920)	0.961 ± 0.054 (0.894–1.000)	0.686 ± 0.088 (0.577–0.795)	0.793 ± 0.069 (0.683–0.902)	0.908 ± 0.107 (0.739–1.000)	0.703 ± 0.136 (0.487–0.918)
2 years	0.892 ± 0.018 (0.869–0.914)	0.904 ± 0.027 (0.871–0.937)	0.756 ± 0.045 (0.700–0.811)	0.762 ± 0.067 (0.679–0.846)	0.778 ± 0.204 (0.525–1.000)	0.775 ± 0.144 (0.596–0.953)
3 years	0.906 ± 0.008 (0.895–0.916)	0.882 ± 0.055 (0.814–0.950)	0.812 ± 0.041 (0.761–0.863)	0.781 ± 0.082 (0.679–0.883)	0.811 ± 0.203 (0.558–1.000)	0.771 ± 0.097 (0.652–0.891)

*Note:* The numbers in parentheses are 95% confidence intervals.

Abbreviations: AUC, area under the ROC curve; Cox‐LASSO, Cox‐least absolute shrinkage and selection operator; Cox‐PH, Cox‐proportional hazards; RSF, random survival forest.

**FIGURE 3:**
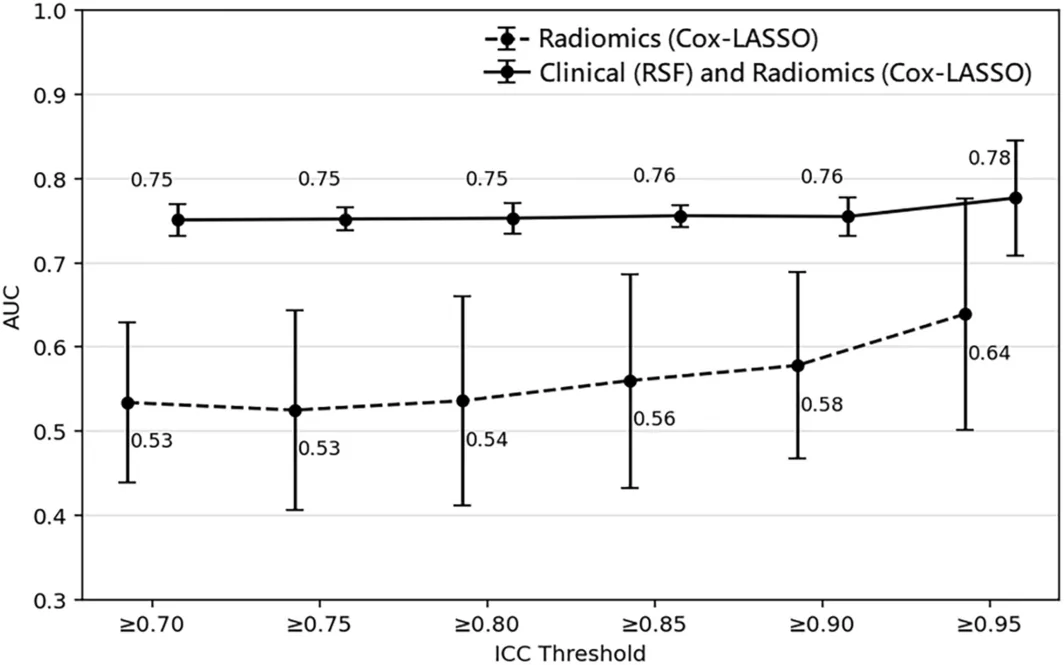
Comparison of radiomics and integration models at ICC thresholds ranging from 0.70 to 0.95.

The comparison of radiomics (Cox‐LASSO) and integration models (RSF clinical and Cox‐LASSO radiomics models) with different ICC thresholds revealed that both the radiomics and integration models with an ICC threshold of ≥ 0.95 demonstrated higher AUC values than those with other ICC thresholds in the testing set (Figure 3).

**FIGURE 4:**
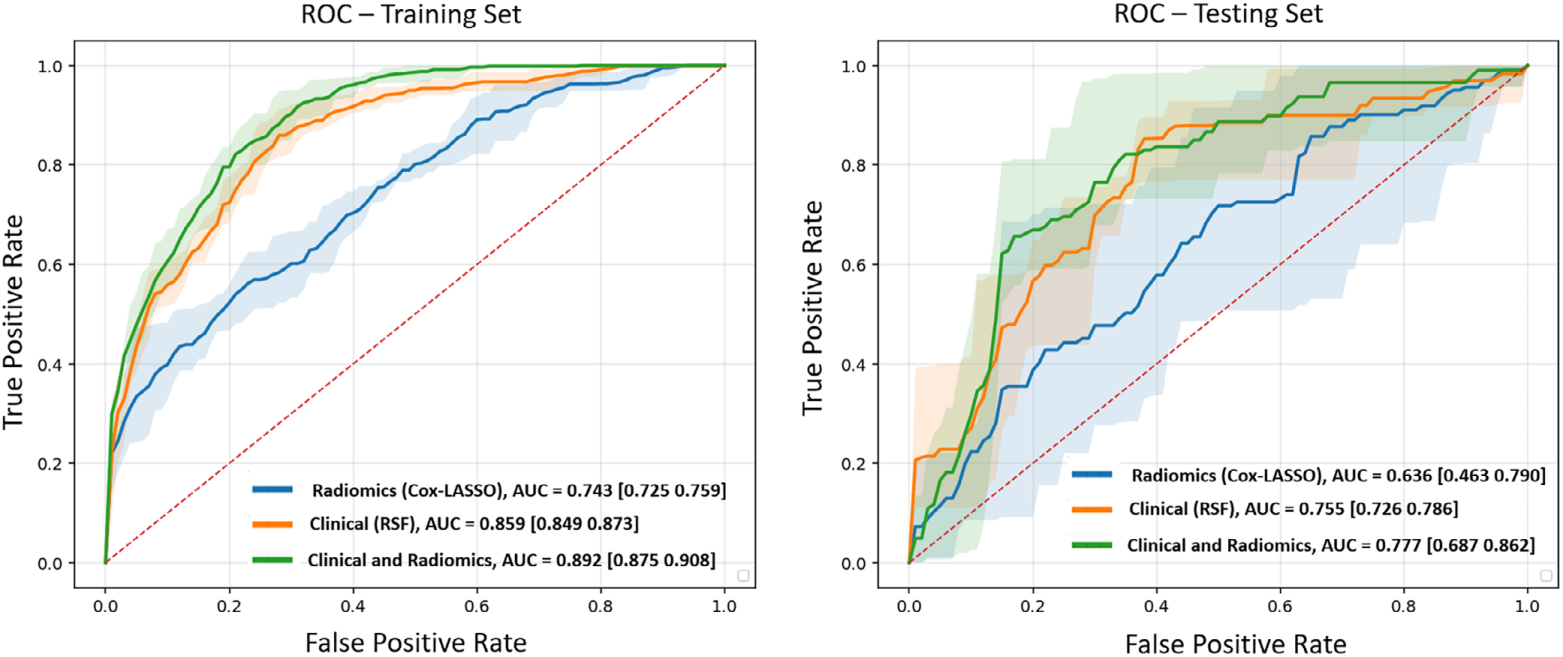
Comparison of ROC curves between the clinical (RSF), radiomics (Cox‐LASSO), and integration models in the training and testing sets within 3 years post‐treatment. The shaded area indicates the 95% confidence interval for the curves. Cox‐LASSO, Cox‐least absolute shrinkage and selection operator; ROC, receiver‐operating characteristic; RSF: random survival forest.

In the training set, the integration model had a mean AUC of 0.892 (95% CI: 0.875–0.908) that surpassed both the clinical model with an AUC of 0.859 (95% CI: 0.849–0.873) and the radiomics model with an AUC of 0.743 (95% CI: 0.725–0.759) in predicting tumor recurrence within 3 years post‐treatment. In the testing set, the comparison also indicated that the integration model with an AUC of 0.777 (95% CI: 0.687–0.862) outperformed both the clinical model with an AUC of 0.755 (95% CI: 0.726–0.786) and the radiomics model with an AUC of 0.636 (95% CI: 0.463–0.790) in predicting tumor recurrence at an ICC threshold of ≥ 0.95 within 3 years post‐treatment (Figure 4).

Moreover, the performance of the two‐algorithm (RSF and Cox‐LASSO) integration model was superior to those of the single‐algorithm integration model trained by combining both clinical and radiomics features using RSF with an AUC of 0.726 (95% CI: 0.708–0.744), Cox‐PH with an AUC of 0.728 (95% CI: 0.694–0.762), and Cox‐LASSO with an AUC of 0.761 (95% CI: 0.748–0.775) algorithms in the testing sets.

**FIGURE 5:**
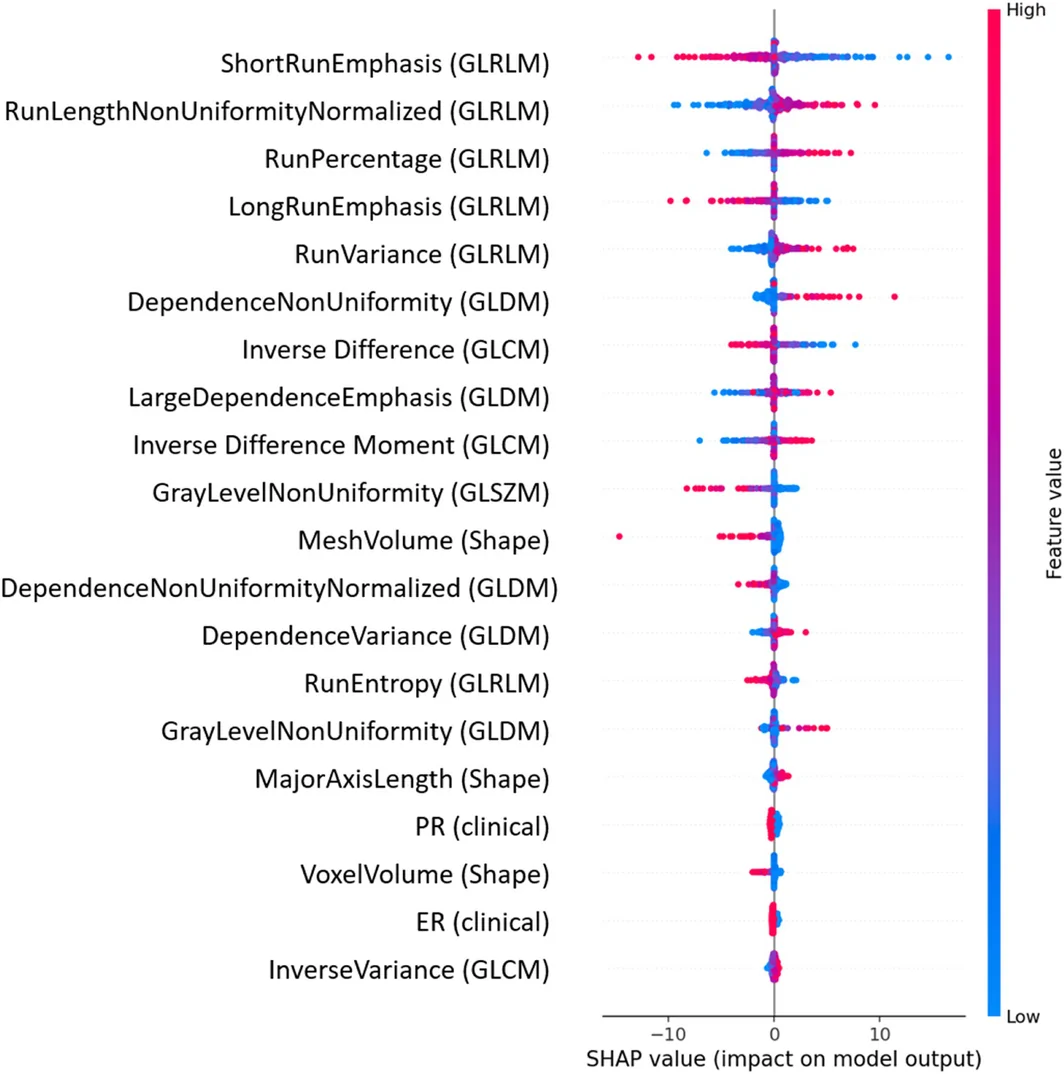
Top 20 important features of the integration model in predicting tumor recurrence for the testing sets.

For the integration model, the SHAP values revealed that the 20 most important features included 18 radiomics features and 2 clinical factors in the testing set (Figure 5).

**FIGURE 6:**
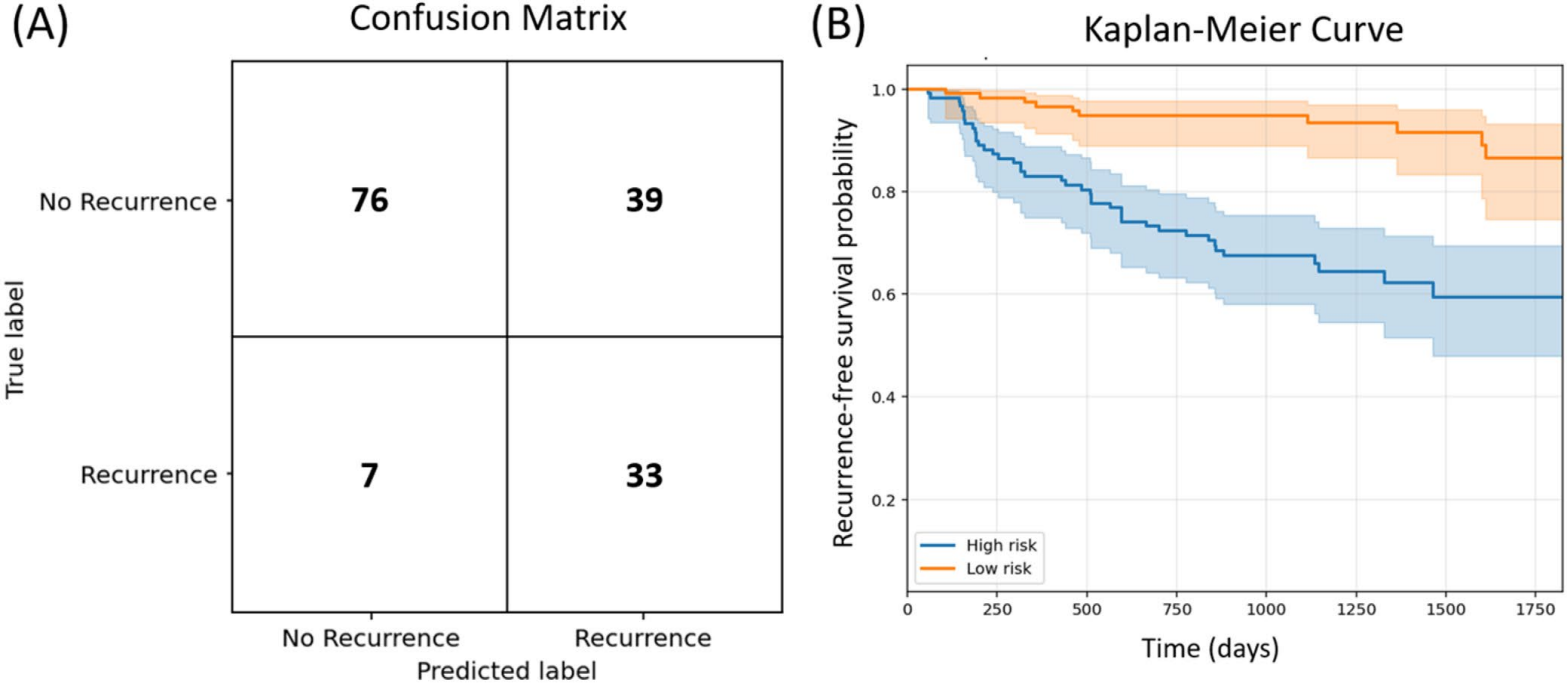
(A) Summed confusion matrix of the prediction results for the testing sets in five‐fold cross‐validation. (B) Kaplan–Meier recurrence‐free survival curves of patients with breast cancer post‐treatment at low and high risks stratified by the integration model for the testing sets. The shaded area indicates the 95% confidence interval for the curves.

The patients in the testing sets were categorized into high‐ and low‐risk groups for tumor recurrence stratified by the integration model in the five‐fold cross‐validation. Kaplan–Meier analysis revealed that patients with low risk of tumor recurrence had significantly different (*p* < 0.001) recurrence‐free survival curves compared to those with high risk (Figure 6).

## 4 Discussion

In patients with breast cancer, the tumor recurrence rate ranges from 5% to 20% after primary treatments, whereas the risk of distant recurrence varies between 13% and 41%, depending on the tumor behavior and staging of the tumor [8, 17, 32, 33]. However, a high risk of tumor recurrence may indicate a poor prognosis for breast cancer. Therefore, predicting tumor recurrence before NST is crucial for adjusting treatment regimens in personalized medicine. To the best of our knowledge, no previous study has developed a machine learning model for pretreatment prediction of breast tumor recurrence using both clinical data and CECT‐based radiomic features in patients undergoing NST. The results of the present study revealed that the integration of clinical and CT‐based radiomics models outperformed both clinical and CT‐based radiomics models in predicting tumor recurrence, indicating that the integration of clinical and radiomics features was helpful for improving the prediction of breast tumor recurrence. Moreover, the Kaplan–Meier analysis revealed that the recurrence‐free survival curves were significantly different between the low‐ and high‐risk groups stratified by the integration model, indicating that the integration model may contribute to the early management of patients with breast cancer with a higher risk of tumor recurrence.

In previous studies, Dasgupta et al. enrolled 83 patients with locally advanced breast cancer and performed machine learning using ultrasound radiomics features to classify patients with and without tumor recurrence. Their results indicated that the support vector machine (SVM) radiomics model performed best with an AUC of 0.76, and the estimated 5‐year recurrence‐free survival was 83% and 54% for non‐recurrence and recurrence groups, respectively [19]. Han et al. recruited 1214 patients with breast cancer who underwent both ultrasound and mammography and categorized them into disease‐free and recurrence groups based on the follow‐up results at 3 years post‐treatment. The combined model (AUC = 0.74) outperformed ultrasound (AUC = 0.68), mammography (AUC = 0.67), ultrasound plus mammography (AUC = 0.70), and clinical (AUC = 0.53) models in the testing cohort [20]. In MRI studies, Lee et al. enrolled 114 patients with (*n* = 28) and without (*n* = 86) locoregional recurrence with propensity score matching. They performed MR‐based machine learning using radiomics features extracted from T2‐weighted images with and without fat suppression and contrast‐enhanced T1‐weighted images with fat suppression. Their results demonstrated that the stacking model of SVM, RF, and logistic regression could achieve an AUC of 0.78 in predicting locoregional recurrence [21]. Yu et al. enrolled 1113 patients with nonmetastatic invasive breast cancer to develop a radiomics deep learning model using features extracted from contrast‐enhanced T1‐weighted and T2‐weighted images. They revealed that the radiomic deep learning model achieved AUCs of 0.98, 0.94, and 0.92 for 1‐, 2‐, and 3‐year recurrence‐free survival, respectively [22]. Moreover, Comes et al. used dynamic contrast‐enhanced MRI of 158 patients with breast cancer with (*n* = 43) and without (*n* = 115) recurrence from two public databases to predict tumor recurrence after neoadjuvant chemotherapy. They developed a hybrid machine learning‐deep learning model and demonstrated that the optimal results could achieve an AUC of 0.83 on the independent testing set [23]. In PET/CT studies, Kawaji et al. developed machine learning models to predict recurrence using pretreatment clinical and ^18^F‐deoxyglucose PET radiomic features in 112 patients with breast cancers. They showed that the combined model RF algorithm (clinical and radiomics data) performed the best with an AUC of 0.99 in the testing set [24].

Conversely, the present study enrolled 235 patients with 237 breast tumors to develop machine learning models using RSF, Cox‐PH, and Cox‐LASSO algorithms with clinical and CT‐based radiomic features. The results revealed that RSF clinical and Cox‐LASSO radiomics models could achieve mean AUCs of 0.755 and 0.636 in predicting tumor recurrence within 3 years in patients after NST, respectively. The integration model achieved a mean AUC of 0.777 in predicting tumor recurrence within 3 years post‐treatment by integrating both clinical and radiomics models. However, the specificity was lower than the sensitivity in the integration model at 1–3 years post‐treatment, likely due to the imbalanced dataset with a low recurrence rate of 23.6%. As a result, the proposed integration model was less effective in filtering out non‐recurrence than in detecting tumor recurrence. CECT scans are considered a routine examination for patients with breast cancer before NST [25]; thus, the findings indicate that the integration of pretreatment clinical and CT‐based radiomics features can effectively detect tumor recurrence after NST.

Moreover, the feature importance using SHAP values for the integration model demonstrated that the top 20 important features comprised 18 radiomics features and 2 clinical features, in which the radiomics features showed higher rankings than clinical features in predicting tumor recurrence. The top radiomics features comprised mainly textural non‐uniformity, tumor volume, and major axis length, whereas the top clinical features comprised ER and PR. Specifically, ShortRunEmphasis, RunLengthNonUniformityNormalized, RunPercentage, LongRunEmphasis, and RunVariance were high‐order textural features that reflect the degree of non‐uniformity in the image within the tumor [28]. These findings indicate that a breast tumor with more tissue heterogeneity and larger tumor size might lead to a higher risk of tumor recurrence than other radiomics features, whereas ER and PR might play more important roles in tumor recurrence than other clinical features in patients with breast cancer after NST.

To ensure reliable radiomic features, the present study employed a semi‐automatic image segmentation method to minimize interobserver variability, achieving a Dice score greater than 0.7, and used the ICC to select radiomic features with good reliability. The comparisons revealed that the radiomics model with an ICC threshold of ≥ 0.95 yielded better performance in predicting tumor recurrence than other ICC thresholds ranging from 0.70 to 0.95. Consistent with previous studies [34], these findings indicated that the Cox‐LASSO algorithm applied to the radiomics model with an ICC threshold of ≥ 0.95 improved the predictive performance of the model. Hence, the final integration model combining the RSF clinical model and Cox‐LASSO radiomics model with an ICC of ≥ 0.95 demonstrated better performance than those with other ICC thresholds.

Several limitations to this study warrant discussion. First, this was a retrospective, single‐center study, and a multicenter cohort study with a larger sample size is warranted to assess the performance of the prediction model using an external dataset. Second, the imbalance in tumor recurrence among the enrolled patients may affect the performance of the prediction model. To address this, SMOTE oversampling was conducted to generate a balanced training dataset and minimize the imbalance issue. Third, the mean follow‐up time in this cohort study was relatively short, at 3.82 ± 2.13 years; thus, the present study only showed the prediction results at 1, 2, and 3 years post‐treatment. A study cohort with a longer follow‐up period may provide better insights into predicting long‐term recurrence‐free survival in patients with breast cancer post‐treatment. Finally, the present study only used the Dice score to evaluate the consistency of tumor segmentation between two readers. The surface‐distance metrics, including Hausdorff distance, median surface distance, normalized surface distance, and average symmetric surface distance, were not compared in the present study.

## 5 Conclusion

The present study developed machine learning models using both clinical and CT‐based radiomic features to predict tumor recurrence in patients with breast cancer after undergoing NST. The results indicated that the clinical model with the RSF algorithm and the radiomics model with the Cox‐LASSO algorithm had better performance than other algorithms. By integrating the clinical and radiomics models, the integration model outperformed both the clinical and radiomics models in predicting tumor recurrence. Further, Kaplan–Meier analysis confirmed that the integration model effectively classified patients into high and low risk for tumor recurrence. In conclusion, the integration of clinical data and radiomics features of the CECT image is a valuable tool for early prediction of tumor recurrence in patients with breast cancer after NST.

## Funding

This research was supported jointly by grants (KMU‐TC114A206 and KMU‐TC115A05‐1) from Kaohsiung Medical University and a grant (NSTC114‐2314‐B‐037‐088) from the National Science and Technology Council of Taiwan.

## Ethics Statement

The local institutional review board of Kaohsiung Medical University Hospital approved this study (protocol code: KMUHIRB‐E(I)‐20200406). The study was conducted in accordance with the Helsinki Declaration, and the need to obtain the informed consent was waived by the local institutional review board of Kaohsiung Medical University Hospital due to the retrospective nature of the study.

## Conflicts of Interest

The authors declare no conflicts of interest.

## Supporting information


**Table S1:** A list of radiomics features used in this study.

## Data Availability

The data that support the findings of this study are available on request from the corresponding author. The data are not publicly available due to privacy or ethical restrictions.
