# Supplementary material for: Pretreatment Prediction of Tumor Recurrence in Breast Cancer After Neoadjuvant Systemic Therapy Using Machine Learning With Clinical and CT Radiomics Features
Source: Kaohsiung J Med Sci. 2026 Aug 26:e70288. Online ahead of print. doi: 10.1002/kjm2.70288 (PMC13519504; doi:10.1002/kjm2.70288)
Supplement: Supplementary file 1 [file KJM2-9999-e70288-s001.docx]

| Shape | **Elongation** |
| --- | --- |
| Shape | **Flatness** |
| Shape | **LeastAxisLength** |
| Shape | **MajorAxisLength** |
| Shape | **Maximum2DDiameterColumn** |
| Shape | **Maximum2DDiameterRow** |
| Shape | **Maximum2DDiameterSlice** |
| Shape | **Maximum3DDiameter** |
| Shape | **MeshVolume** |
| Shape | **MinorAxisLength** |
| Shape | **Sphericity** |
| Shape | **SurfaceArea** |
| Shape | **SurfaceVolumeRatio** |
| Shape | **VoxelVolume** |
| First-order | **10Percentile** |
| First-order | **90Percentile** |
| First-order | **Energy** |
| First-order | **Entropy** |
| First-order | **InterquartileRange** |
| First-order | **Kurtosis** |
| First-order | **Maximum** |
| First-order | **MeanAbsoluteDeviation** |
| First-order | **Mean** |
| First-order | **Median** |
| First-order | **Minimum** |
| First-order | **Range** |
| First-order | **RobustMeanAbsoluteDeviation** |
| First-order | **RootMeanSquared** |
| First-order | **Skewness** |
| First-order | **TotalEnergy** |
| First-order | **Uniformity** |
| First-order | **Variance** |
| GLCM | **Autocorrelation** |
| GLCM | **ClusterProminence** |
| GLCM | **ClusterShade** |
| GLCM | **ClusterTendency** |
| GLCM | **Contrast** |
| GLCM | **Correlation** |
| GLCM | **DifferenceAverage** |
| GLCM | **DifferenceEntropy** |
| GLCM | **DifferenceVariance** |
| GLCM | **InverseDifference** |
| GLCM | **InverseDifferenceMoment** |
| GLCM | **InverseDifferenceMomentNormalized** |
| GLCM | **InverseDifferenceNormalized** |
| GLCM | **InformationalMeasureCorrelation 1** |
| GLCM | **InformationalMeasureCorrelation 2** |
| GLCM | **InverseVariance** |
| GLCM | **JointAverage** |
| GLCM | **JointEnergy** |
| GLCM | **JointEntropy** |
| GLCM | **MaximalCorrelationCoefficient** |
| GLCM | **MaximumProbability** |
| GLCM | **SumAverage** |
| GLCM | **SumEntropy** |
| GLCM | **SumSquares** |
| GLDM | **DependenceEntropy** |
| GLDM | **DependenceNonUniformity** |
| GLDM | **DependenceNonUniformityNormalized** |
| GLDM | **DependenceVariance** |
| GLDM | **GrayLevelNonUniformity** |
| GLDM | **GrayLevelVariance** |
| GLDM | **HighGrayLevelEmphasis** |
| GLDM | **LargeDependenceEmphasis** |
| GLDM | **LargeDependenceHighGrayLevelEmphasis** |
| GLDM | **LargeDependenceLowGrayLevelEmphasis** |
| GLDM | **LowGrayLevelEmphasis** |
| GLDM | **SmallDependenceEmphasis** |
| GLDM | **SmallDependenceHighGrayLevelEmphasis** |
| GLDM | **SmallDependenceLowGrayLevelEmphasis** |
| GLRLM | **GrayLevelNonUniformity** |
| GLRLM | **GrayLevelNonUniformityNormalized** |
| GLRLM | **GrayLevelVariance** |
| GLRLM | **HighGrayLevelRunEmphasis** |
| GLRLM | **LongRunEmphasis** |
| GLRLM | **LongRunHighGrayLevelEmphasis** |
| GLRLM | **LongRunLowGrayLevelEmphasis** |
| GLRLM | **LowGrayLevelRunEmphasis** |
| GLRLM | **RunEntropy** |
| GLRLM | **RunLengthNonUniformity** |
| GLRLM | **RunLengthNonUniformityNormalized** |
| GLRLM | **RunPercentage** |
| GLRLM | **RunVariance** |
| GLRLM | **ShortRunEmphasis** |
| GLRLM | **ShortRunHighGrayLevelEmphasis** |
| GLRLM | **ShortRunLowGrayLevelEmphasis** |
| GLSZM | **GrayLevelNonUniformity** |
| GLSZM | **GrayLevelNonUniformityNormalized** |
| GLSZM | **GrayLevelVariance** |
| GLSZM | **HighGrayLevelZoneEmphasis** |
| GLSZM | **LargeAreaEmphasis** |
| GLSZM | **LargeAreaHighGrayLevelEmphasis** |
| GLSZM | **LargeAreaLowGrayLevelEmphasis** |
| GLSZM | **LowGrayLevelZoneEmphasis** |
| GLSZM | **SizeZoneNonUniformity** |
| GLSZM | **SizeZoneNonUniformityNormalized** |
| GLSZM | **SmallAreaEmphasis** |
| GLSZM | **SmallAreaHighGrayLevelEmphasis** |
| GLSZM | **SmallAreaLowGrayLevelEmphasis** |
| GLSZM | **ZoneEntropy** |
| GLSZM | **ZonePercentage** |
| GLSZM | **ZoneVariance** |
| NGTDM | **Busyness** |
| NGTDM | **Coarseness** |
| NGTDM | **Complexity** |
| NGTDM | **Contrast** |
| NGTDM | **Strength** |

GLCM: Gray-Level Co-occurrence Matrix

GLDM: Gray Level Dependence Matrix

GLRLM: Gray-Level Run-Length Matrix

GLSZM: Gray-Level Size Zone Matrix

NGTDM: Neighborhood Gray-Tone Difference Matrix
